# Nonlinear effect of carrier drift on the performance of an n-type ZnO nanowire nanogenerator by coupling piezoelectric effect and semiconduction

**DOI:** 10.3762/bjnano.9.183

**Published:** 2018-07-04

**Authors:** Yuxing Liang, Shuaiqi Fan, Xuedong Chen, Yuantai Hu

**Affiliations:** 1 Department of Mechanics, Hubei Key Laboratory of Engineering Structural Analysis and Safety Assessment, Huazhong University of Science and Technology, Wuhan 430074, China; 2 State Key Laboratory of Digital Manufacturing Equipment & Technology, Huazhong University of Science and Technology, Wuhan 430074, China

**Keywords:** carrier drift, crystallogrpahic *c*-axis, piezoelectric potential, semiconductor, zinc oxide (ZnO)

## Abstract

In piezoelectric semiconductors, electric fields drive carriers into motion/redistribution, and in turn the carrier motion/redistribution has an opposite effect on the electric field itself. Thus, carrier drift in a piezoelectric semiconducting structure is essentially nonlinear unless the induced fluctuation of carrier concentration is very small. In this paper, the nonlinear governing equation of carrier concentration was established by coupling both piezoelectric effect and semiconduction. A nonlinear carrier-drift effect on the performance of a ZnO nanogenerator was investigated in detail and it was elucidated that carrier motion/redistribution occurs in the ZnO nanowire (ZNW) cross section while there is no carrier motion in the axial direction. At the same time, we noted that the amplitude of boundary electric charge grows with increasing deformation, but the peaks of boundary electric charge do not appear at the cross-section endpoints. Thus, in order to effectively improve the performance of the ZNW nanogenerator, the effect of electrode configuration on the piezoelectric potential difference and output power was analyzed in detail. The electrode size for the optimal performance of a ZnO nanowire generator was proposed. This analysis that couples electromechanical fields and carrier concentration as a whole has some referential significance to piezotronics.

## Introduction

An acoustic wave propagating in piezoelectric semiconductors usually stimulates electric fields that bring charge carriers into motion, and conversely, the carrier motion will produce an opposite effect on the electric fields and the acoustic wave itself [1–4]. This kind of interaction between an acoustic wave and carriers in piezoelectric semiconductors is called the acoustoelectric effect, which is a special case of a more general phenomenon, called wave–particle drag [4–5]. Obviously, acoustoelectric coupling of piezoelectric semiconductors can be used to develop many new microelectronic devices with modern functions, for example piezoelectric field-effect transistors [6–11], piezoelectric charge-coupled devices [12–15], piezoelectric chemical sensors [16–17], and nanogenerators made of vertically aligned ZnO nanowires [18–27]. The principle of nanogenerators is that the piezoelectric potential produced by the piezoelectric effect can produce a current in an external circuit when the ZNW is deformed. Specifically, a transversely applied force makes the nanowire bent when an atomic force microscopy tip scans over the top of the nanowire. The electromechanical coupling converts mechanical energy into electric energy [28–29]. A piezoelectric potential is built inside the nanowire with the stretched side being positively charged and the compressed side being negatively charged. At the same time, a Schottky barrier formed between the AFM tip and the nanowire and the piezoelectric potential will not disappear as long as the mechanical stress is maintained. This potential can be made use of to generate an electrical current [30].

Recently, Fan et al. studied the linear solutions of electromechanical quantities in a bent ZNW under the assumption of a small fluctuation of the carrier concentration [31]. Electric fields are proven to be independent of the axial position along the *c*-axis except near the end regions, and carrier motion/redistribution is proven to occur along the cross section. Because the semiconduction in ZNWs results in some electric leakage, a smaller initial carrier concentration is suggested to be more proper for energy-harvesting from a bent ZNW [31]. Because a small fluctuation of the carrier concentration implies a small deformation, a very low output of ZNW nanogenerators occurs under that situation. The force necessarily to obtain a good performance of the nanogenerator will certainly result in large variations of carrier concentration [30]. Thus, we abandon the assumption of small carrier-concentration fluctuations and establish the corresponding nonlinear governing equation of carrier concentration in this paper. The nonlinear accumulation of n-type carriers on one side of the ZNW cross section is shown in detail. Distribution characteristics of carrier concentration and electric potential in the cross section are discussed. Both the boundary electric charge and the boundary electric potential difference are calculated in depth. It is found that the amplitude of boundary electric charge always grows with increasing deformation, but the peaks of boundary electric charge do not appear directly at the cross-section endpoints. For harvesting a bent beam-like structure, the output electrodes are usually placed near the two cross section endpoints [30,32–33]. To improve the performance of a ZNW nanogenerator, the electrode configuration should be optimized with regard to piezoelectric potential difference and output power. We also carry out a detailed analysis on the effect of the electrode configuration.

## Nonlinear Governing Equation of Carrier Concentration for a Bent ZNW

**Figure 1:**
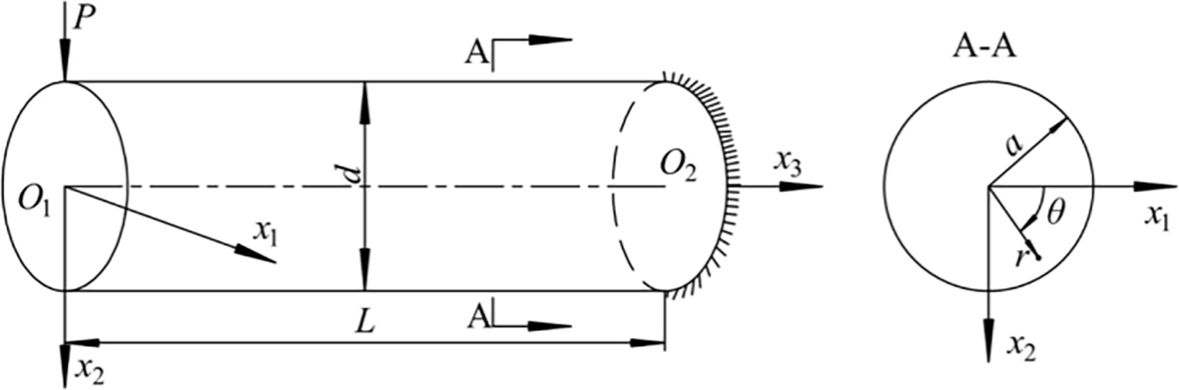
A circular ZNW cantilever exposed to a force *P* at the free end.

In a bent ZnO nanowire as shown in Figure 1, elastic fields can be solved in advance by using the irrotationality of static electric fields [31]. The two in-plane electric field components, *E*_1_ and *E*_2_, can be proven only to depend on *x*_1_ and *x*_2_, while the out-of-plane component *E*_3_ is zero. The electric displacements, **D** = (*D*_1_, *D*_2_, *D*_3_)^T^, in the ZNW are:

[1]
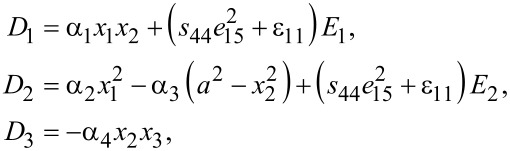


with

[2]
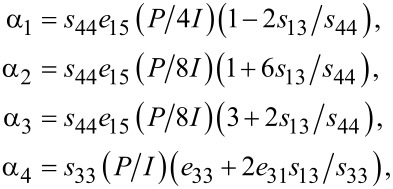


where 
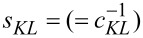
 are the compliance coefficients with *c_KL_* being the elastic constants; ε*_ij_* being the dielectric constants and *e_iL_* being the electromechanical coupling coefficients. *K*, *L* = 1, 2,…, 6; *i, j* = 1, 2, 3; 
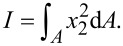
 The cross section of a bent ZnO nanowire is assumed to be circular in our analysis, which was also assumed by, for example, Gao and Wang [26–27], Henneghien et al. [34], and Maslov and co-workers [35]. In particular, Henneghien et al. have pointed out that circular and hexagonal nanowires exhibit the same behavior if the ZnO nanowire sections of each structure have the same area.

Linear solutions were obtained for small fluctuations of carrier concentration in a bent n-type ZnO nanowire under a force *P* = 0.7 nN at the end of the nanowire [31]. However, small carrier concentration fluctuations, 
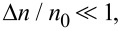
 where Δ*n* = *n* − *n*_0_, imply a very low output of the ZNW generator under consideration. Here, *n* and *n*_0_ stand for the actual and the initial carrier concentration, respectively. *P* should be enlarged for obtaining a better performance of the nanogenerator. Electric fields appearing in piezoelectric semiconductors will move/redistribute carriers and, in turn, the motion/redistribution of carriers will have an influence on the electric fields. This indicates that the first carrier drift term in the electric current expression, *J_i_* = *qn*μ*_ij_E_j_* + *q*κ*_ij_n_,j_*, is essentially nonlinear. Since a ZNW with the crystallographic *c*-axis along the *x*_3_-direction is transversely isotropic, the electron mobility μ*_ij_* and the diffusion coefficients κ*_ij_* can be written as

[3]
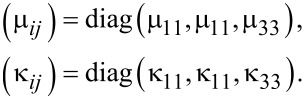


The two in-plane components of the electric field can thus be obtained from *J_i_* = *qn*μ*_ij_E_j_* + *q*κ*_ij_n_,j_* = 0, the null-current condition, as follows with ζ = κ_11_/μ_11_:

[4]
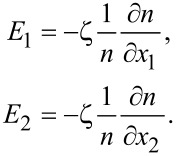


Substituting Equation 4 into Equation 1 and then into the Gauss law, 
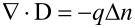
, yields the nonlinear governing equation of carrier concentration 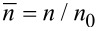 as

[5]
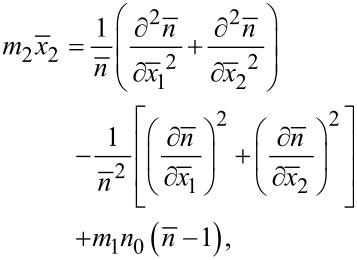


where 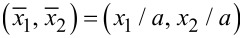 and

[6]
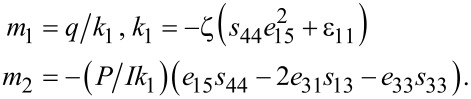


In the vacuum outside the ZNW cross-section, the electric potential 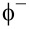 should satisfy

[7]
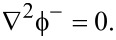


The point of zero electric potential is set at infinity, i.e., 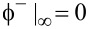. The continuous conditions of normal electric displacement 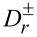 and electric potential 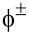 at the boundary Ω, *r* = *a*, of the ZNW cross section require

[8]
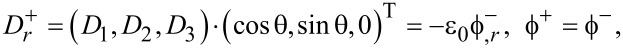


where 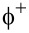 refers to the electric potential within the ZNW cross section, and *r* and θ stand for the radial and the tangential coordinate, respectively, as shown in Figure 1. ε_0_ is the dielectric coefficient of the vacuum.

## Results and Discussion

We calculate carrier concentration fluctuation, piezoelectric potential, electric fields, boundary charges by using the finite element method for *P* from 0.7 nN to 80 nN, where the ZnO nanowire has a diameter *d* = 50 nm and its *c*-axis is oriented along the *x*_3_-direction. The material constants are given below in Equation 9 [36–41] with ε_0_ = 8.8542·10^−12^ F/ m.

[9]
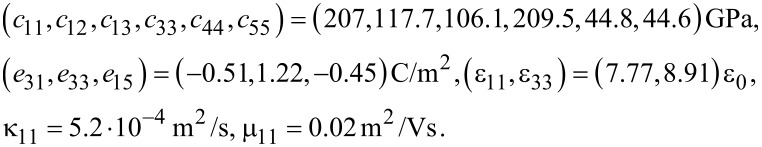


**Figure 2:**
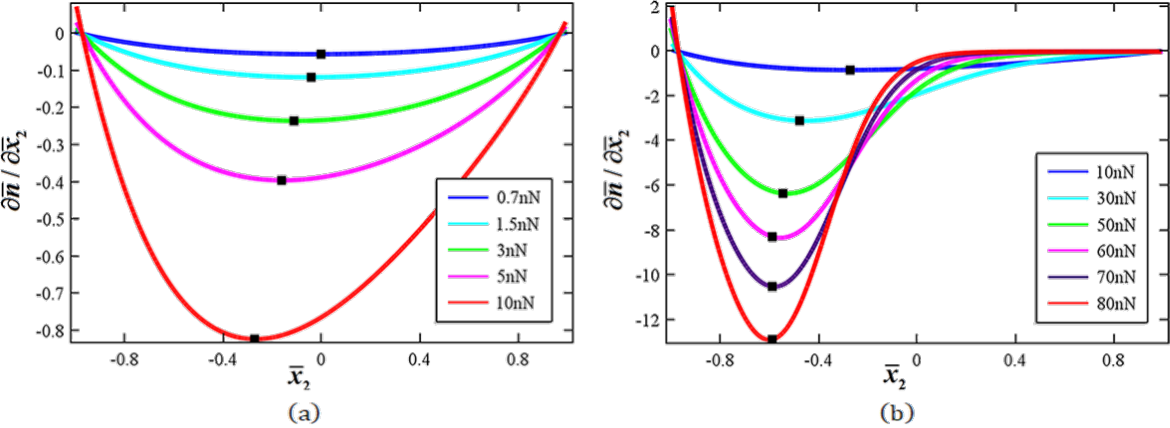
Nonlinearity as a result of carrier drift for *n*_0_ = 1.0·10^23^m^−3^ as a function of the end force *P*. a) P = 0.7, 1.5, 3.0, 5.0 and 10.0 nN; b) *P* = 10, 30, 50, 60, 70 and 80 nN.

For a bent n-type ZNW in the linear regime, a positive piezoelectric potential appears at the stretched side and negative piezoelectric potential appears at the compressed side [26–31], i.e., the electric potential is high at 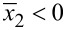 and low at 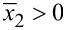. When the end force *P* increases, n-type carriers will be driven to drift upwards with an accumulation at the stretched side, Δ*n*/*n*_0_, becoming so large that the linear balance regime collapses. Non-uniformity in carrier concentration accompanied by drift will bring about also diffusion. These two effects of drift and diffusion will achieve a new equilibrium at every step of increase in *P*. A higher accumulation of carriers in 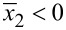 results in a stronger nonlinearity. Figure 2a shows the nonlinearity manifesting itself from carrier drift with *n*_0_ = 1.0·10^23^m^−3^ for *P* = 0.7, 1.5, 3.0, 5.0 and 10.0 nN. *P* = 0.7 nN corresponds to the linear case of which the largest variance of carrier concentration, 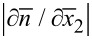, appears along the neutral axis [31]. There are two factors to affect 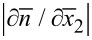: mechanical shear deformations and accumulation of electric carriers. In general, shear deformations result in in-plane electric fields, which lead to carrier drift, while carrier accumulation results in diffusion. Because Δ*n*/*n*_0_ is very small in the whole ZNW cross section in the linear regime, the largest variance rate of carrier concentration appears along the neutral axis because of the strongest shear deformation there. With increasing end force, the carrier accumulation increases such that there is a stronger nonlinear drift effect on 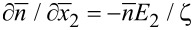. The carrier concentration gradually grows in the upper portion and reduces in the lower portion, which yields 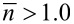 for 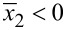 and 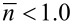 for 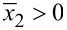. Hence, at larger *P*, 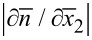 becomes larger for 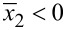 and smaller for 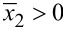 and the position of the largest variance 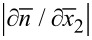 gradually shifts upwards, as shown in Figure 2a. When *P* becomes very large (Figure 2b, *P* = 10, 30, 50, 60, 70 and 80 nN for *n*_0_ = 1.0·10^23^m^−3^), the position of the largest variance shifts upwards even more, implying that most of the carriers are accumulated in the upper cross section.

**Figure 3:**
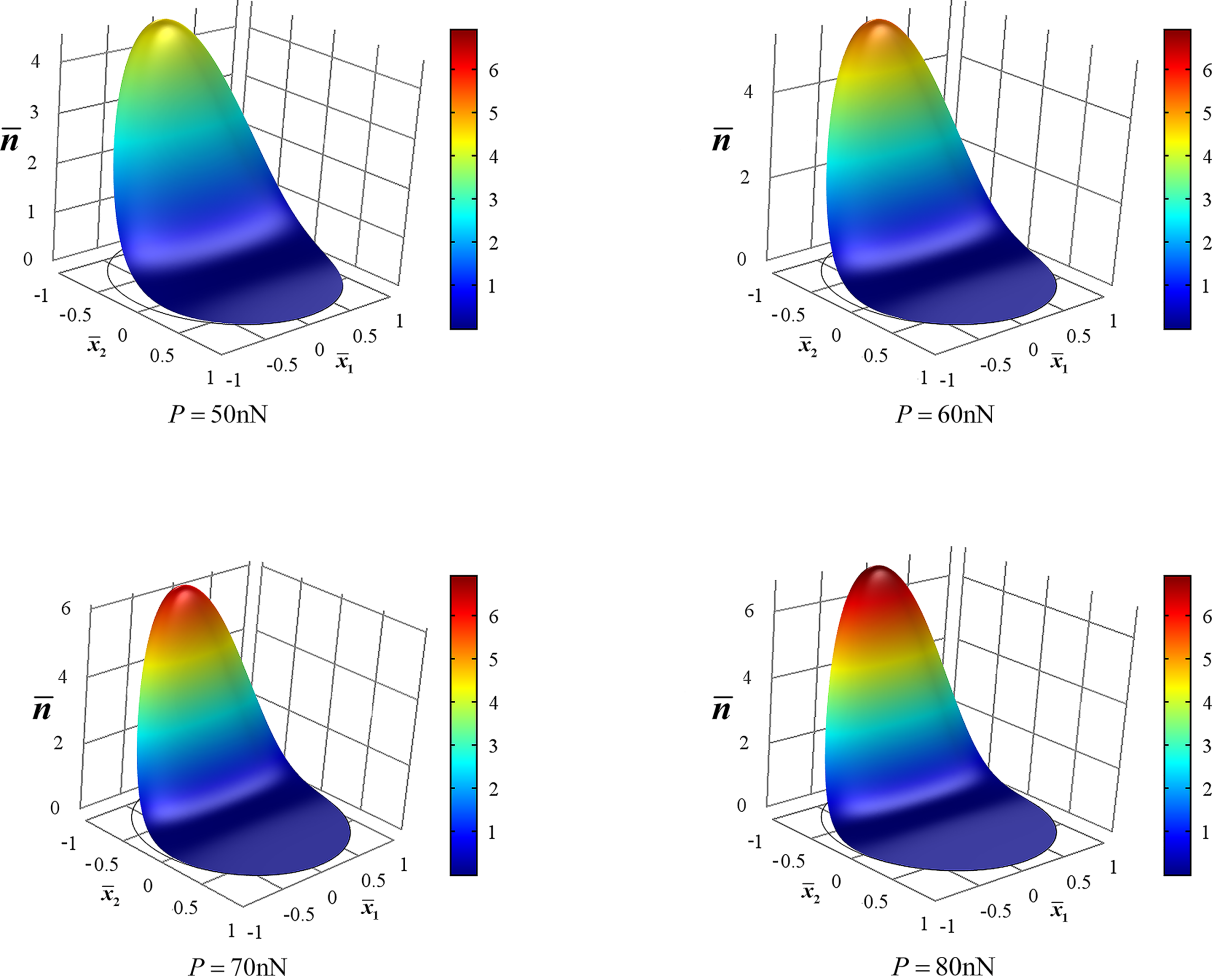
Carrier distribution in the ZNW cross section for *P* = 50, 60, 70 and 80 nN.

Figure 3 shows the carrier distribution in the ZNW cross section for *P* = 50, 60, 70 and 80 nN. It is easy to find numerically that 
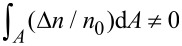
. Given the charge-balance condition this results needs to be explained. It is very obvious that there is no drift and diffusion of carriers along the axial *x*_3_-direction due to *E*_3_ = 0. Carrier drift and diffusion only occur along the cross section. In thermal equilibrium, the Fermi energy level Σ_f_ must be consistent in the cross section with the carrier concentration satisfying

[10]
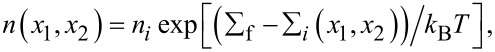


where *n_i_* is the intrinsic carrier concentration of ZnO; Σ*_i_*(*x*_1_, *x*_2_) stands for the intrinsic energy level that can be affected by the electric potential field; *k*_B_ is the Boltzmann constant and *T* is the temperature (300 K in our analysis). *n*_0_ = *n_i_*·exp[(Σ_f_ − Σ*_i_*(0))/*k*_B_*T*], Σ*_i_*(0) is the initial intrinsic energy level, which is constant in the whole cross section under *P* = 0. When the ZNW is bent by a nonzero end force *P*, Σ*_i_*(*x*_1_,*x*_2_) becomes alterable in the cross section in terms of the electric potential field, which turns Equation 10 into

[11]
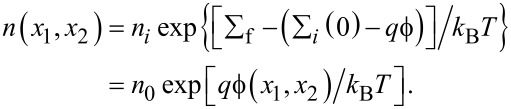


**Figure 4:**
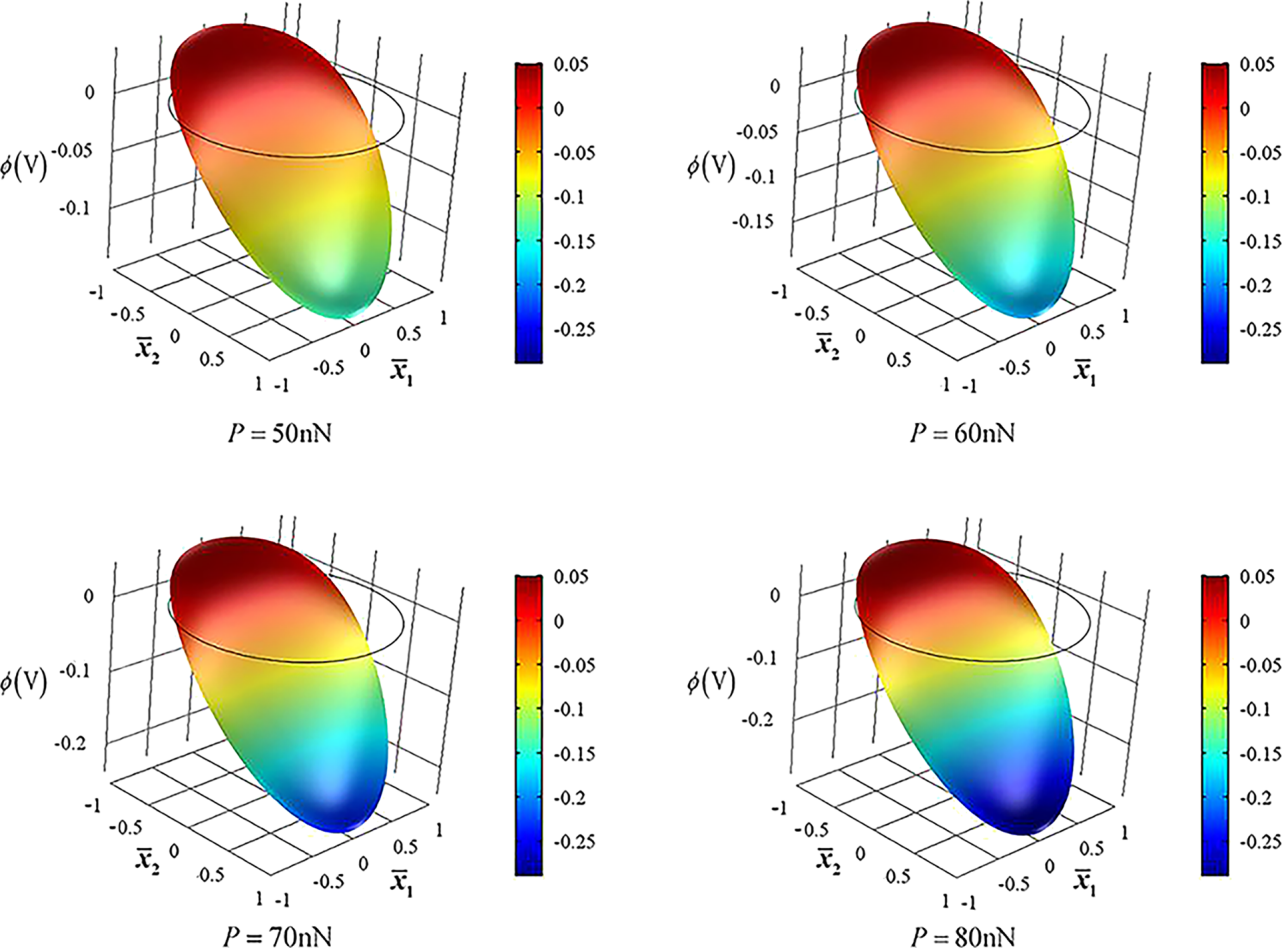
Distribution of electric potential in the ZNW cross section for *P* = 50, 60, 70 and 80 nN.

Equation 11 indicates an exponential relationship between carrier concentration and electric potential in the cross section. Figure 4 shows the distribution of the electric potential in the ZNW cross section for *P* = 50, 60, 70 and 80 nN, which can also be obtained directly from Figure 3 by using Equation 11. In the electric potential field, 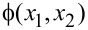, all n-type carriers in the cross section obtain additional electric potential energy, 
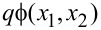
. This potential energy increases/reduces the energy of electrons there, and thus, increases/decreases the number of the n-type carriers according to Equation 11, i.e., it is the appearance of additional electric potential energy induced by the electric potential field that results in 
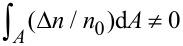
.

**Figure 5:**
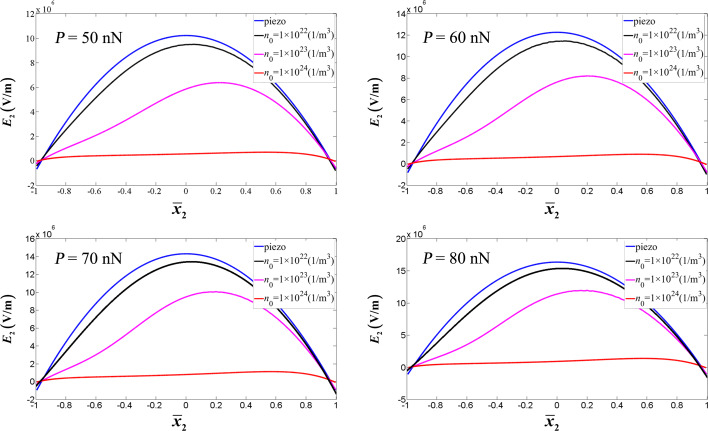
Effect of initial carrier concentration *n*_0_ on the electric field *E*_2_ along the *x*_2_-axis.

It follows from Figure 4 that there is a strong electric potential gradient along the *x*_2_-direction, and the maximal electric potential difference is between the two endpoints of the *x*_2_-axis. Furthermore, the maximal positive potential amplitude is much lower than the maximal negative potential amplitude. In a bent piezoelectric semiconducting beam, two shear deformations produce two in-plane electric field components, *E*_1_ and *E*_2_. *E*_1_ is induced by the shear strain *S*_5_ in the *x*_1_–*x*_3_ plane and *E*_2_ is induced by the shear strain *S*_4_ in the *x*_2_–*x*_3_ plane. When *P* acts along the *x*_2_-direction, σ_5_(τ_13_) is very small, and so is *S*_5_. Thus, both σ_5_ and *S*_5_ produce negligible influence on electric field, carrier concentration and electric potential. *S*_4_ is the primary deformation component to induce the electric field *E*_2_ and carrier redistribution. We show in Figure 5 the effect of *n*_0_ on *E*_2_ in order to understand why the maximal positive potential amplitude is much less than the maximal negative potential amplitude [27]. For comparison, we have also included the electric field *E*_2_ induced only by the piezoelectric effect of a ZNW without taking into account semiconduction (marked as “piezo” in the following figures). Independently of the deformation, in a piezoelectric insulator ZNW (without semiconduction) its Fermi energy level is always at the center of the forbidden band. Hence, the electric field *E*_2_ induced only by the piezoelectric characteristics is symmetrical with regard to the neutral axis, as shown in Figure 5. After n-type doping, the Fermi level moves upwards from the center of the forbidden band. The deformation-induced electric field induces motion/redistribution of carriers: the positive electric field *E*_2_ drives the carriers upwards, i.e., the bottom portion, 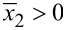, loses carriers and the upper portion, 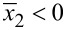, accumulates carriers. This motion/redistribution of carriers will in turn decrease the positive electric field *E*_2_. Moreover, *E*_2_ is reduced more at 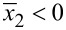 than at 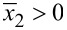. It can be found from Figure 3 that 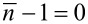 occurs at 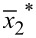 ≈ −0.236, −0.273, −0.309 and −0.338 for *P* = 50, 60, 70 and 80 nN, respectively, with *n*_0_ = 1.0·10^23^ m^−3^. It should be noted that at 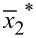, 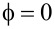 and the intrinsic energy level is Σ*_i_*(0). Because 
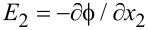
 is reduced more in the region 
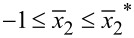
 than in 
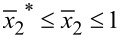
, as shown in Figure 5, the amplitude of the positive electric potential at 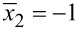 becomes much smaller than the amplitude of the negative electric potential at 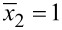. For example, for *P* = 80 nN, the maximal positive electric potential at 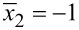 is about 0.05 V and the maximal negative potential at 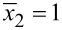 is about 0.3 V, which is in agreement with the experimental results described on page 36 of [30].

**Figure 6:**
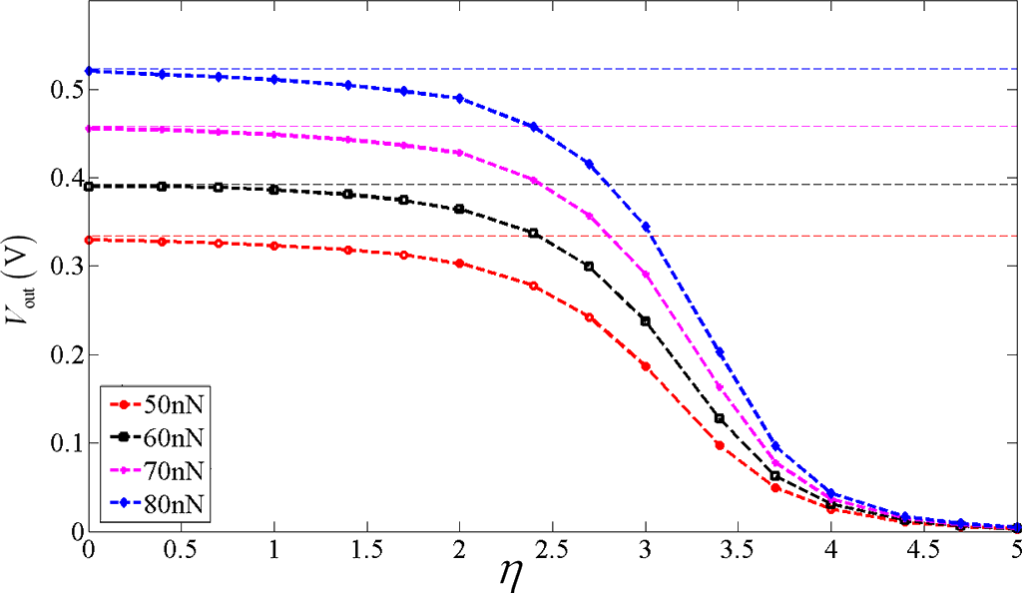
Output voltage *V*_out_ between the two endpoints of the cross section of a bent ZNW as a function of the initial carrier concentration.

We now introduce η = *n*_0_/*n*′ with *n*′ = 1·10^20^ m^−3^. Figure 6 shows effect of the initial carrier concentration *n*_0_ on the output voltage 
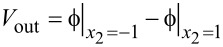
 between the two endpoints of the *x*_2_-axis of a bent ZNW cross section for *P* = 50, 60, 70 and 80 nN, respectively. We have included in Figure 6 four horizontal lines for comparison, corresponding to the four output voltages calculated only from the pure piezoelectric effect without taking semiconduction into account. For a low initial carrier concentration, for example η < 0.5, the Fermi energy level is a little bit above the center of the forbidden band. Thus, the output voltage is very similar to that of the purely piezoelectric effect. For higher initial carrier concentrations, e.g., η > 4.5, the Fermi energy level is far higher than the center of the forbidden band. Hence, the output voltage becomes very low. This phenomenon indicates again that a smaller initial carrier concentration is more proper for energy harvesting with a bent ZNW [31].

**Figure 7:**
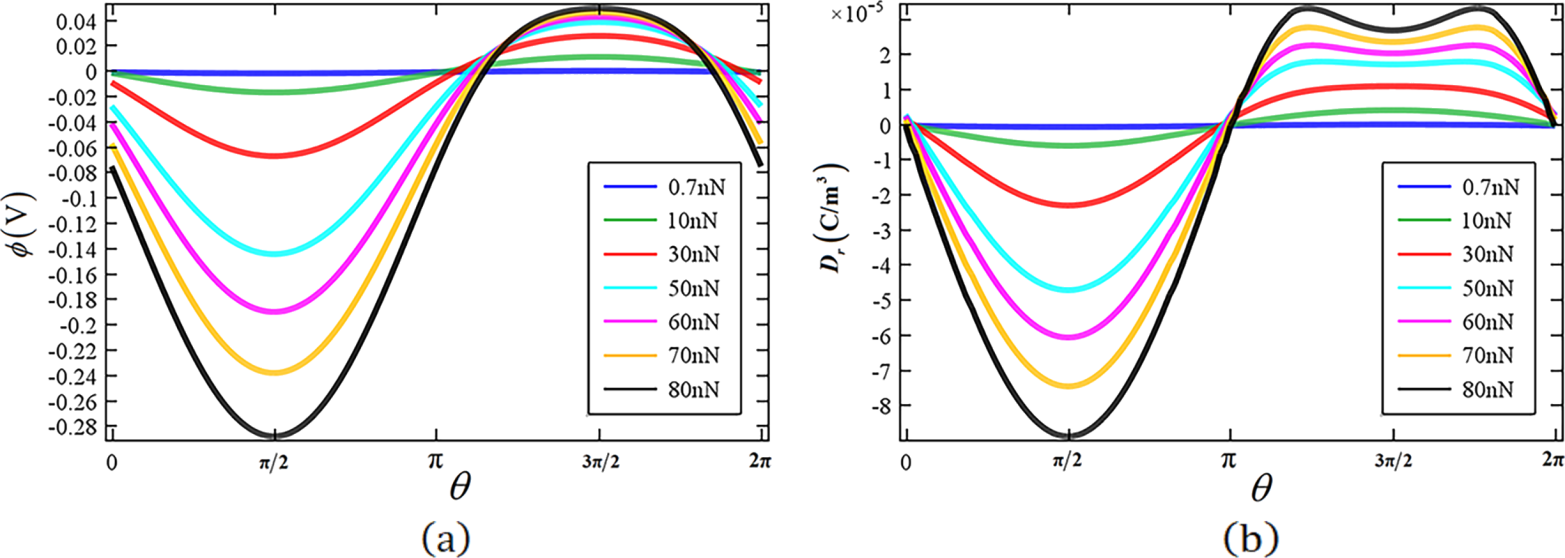
(a) Boundary electric potential 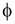 and (b) boundary electric displacement *D_r_* at Ω for different end forces *P* = 0.7, 10, 30, 50, 60, 70 and 80 nN.

Figure 7a shows the distributions of the boundary electric potential for different end forces with *n*_0_ = 1·10^23^ m^−3^. The boundary electric potential reaches the minimum and the maximum at θ = π/2 and θ = 3π/2, respectively, which indicates that the maximal electric potential difference *V*_out_ occurs between the endpoints at top and bottom. Furthermore, *V*_out_ increases with increasing *P*. Figure 7b shows the boundary electric displacement at Ω as a function of the end force. We note that with an increase in end force *P*, the maximal boundary electric displacement does no longer appear at the upper endpoint of the *x*_2_-axis. Instead, there are two peaks in the region π < θ < 2π with a certain angular deviation from the endpoint. The appearance of this phenomenon is due to the excessive nonlinear accumulation of carriers in the upper part of the cross section. The boundary charge is quite large between these two peaks and should be collected. Thus, the design of an effective electrode configuration becomes a significant issue in order to improve the output of a ZnO nanogenerator.

**Figure 8:**
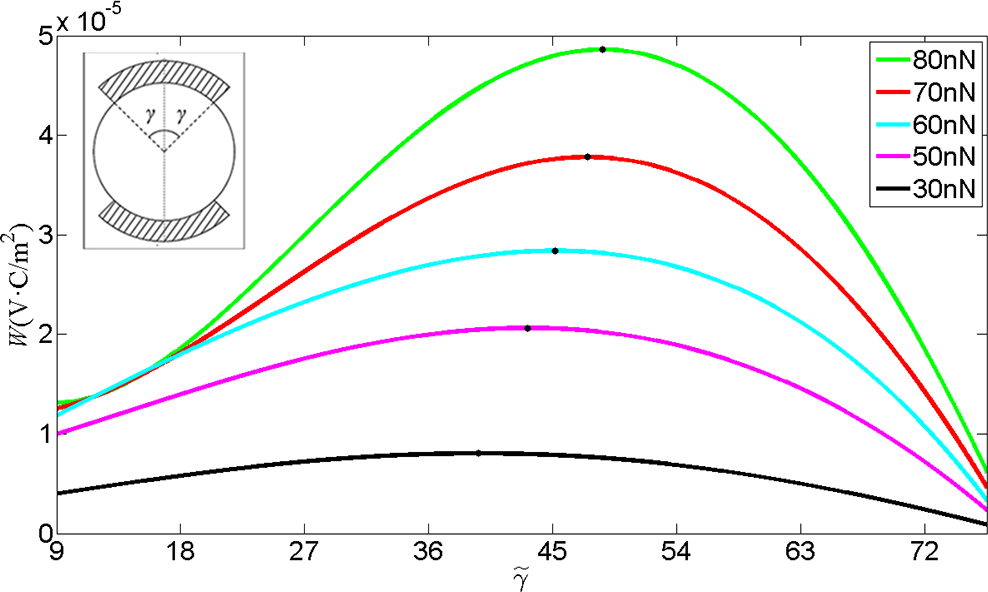
*W* as a function of the electrode configuration for different end forces.

Consider the electrode configuration in the inset of Figure 8. We repeat the numerical calculation by dividing the boundary into two parts: one is the continuous boundary (outside the electrodes) and the other is constant electric potential boundary (inside the electrodes). In this situation, the electric charge *Q_e_* at the electrode can be obtained [42–43] through the integral of the boundary electric displacement *D_r_* over the distributed surface 3π/2 − γ < θ < 3π/2 + γ,

[12]
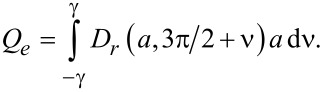


The current flowing through this electrode is 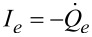, which indicates that the output current *I_e_* for a harmonic vibration is directly proportional to the amplitude of the boundary electric charge *Q_e_*. Thus, for convenience we define the quantity *W*,

[13]
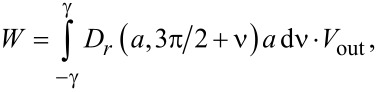


which is directly proportional to the output power of the ZNW generator. 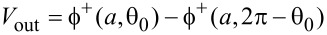 stands for the electric potential difference between the two electrodes and θ_0_ = γ + 3π/2. Figure 8 shows *W* as a function of 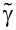, with 
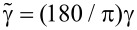
. With *P* changing from 30 to 80 nN, the peak point of *W* moves from θ_0_ = 1.72π (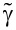 ≈ 40°) to θ_0_ = 1.77π (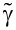 ≈ 49°). Obviously, a flare angle of 2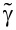 ≈ 98° of the two electrodes is suitable for all loadings below 80 nN. This electrode configuration provides an optimal output of the nanogenerator.

**Figure 9:**
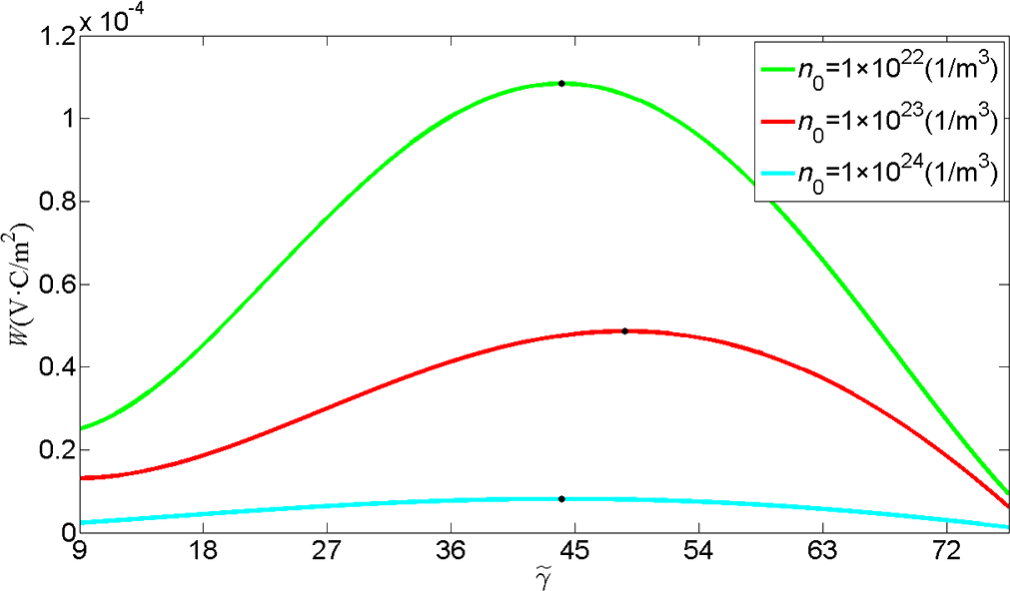
*W* as a function of the electrode configuration for different initial carrier concentrations.

In addition, we show the effect of the initial carrier concentration on the optimal electrode configuration in Figure 9 for *n*_0_ = 1·10^22^, 1·10^23^ and 1·10^24^ m^−3^. Obviously, a smaller initial carrier concentration is corresponding to a higher energy harvest. It is readily understood that semiconduction results in some electric leakage, and thus reduces the output power. For different initial carrier concentrations, electrode configurations with flare angles of 2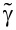 ≈ 88°, 98° and 88° are suitable for a ZNW generator with *n*_0_ = 1·10^22^, 1·10^23^ and 1·10^24^ m^−3^, respectively.

## Conclusion

Nonlinear solutions for carrier concentration, electric field and electric potential in a bent ZNW are obtained without the assumption of small fluctuations of carrier concentration. As the bending deformation increases, carriers are gradually accumulated in the stretched portion of the ZNW, and the boundary charge is greatly increased. It is found that the electrode configuration will have a large influence on the output performance of a bent ZNW generator. Thus it is of significance to design an electrode configuration for an optimal output. Both the analysis technique and the obtained results are useful in the design of piezotronics and piezo-phototropic devices and the corresponding applications.
